# Targeting the leptin receptor promotes MDA-MB-231 cells’ metabolic reprogramming and malignancy: the role of extracellular vesicles derived from obese adipose tissue

**DOI:** 10.3389/fonc.2025.1568524

**Published:** 2025-05-23

**Authors:** Carol Costa Encarnação, Carolinne Souza Amorim, Victor Aguiar Franco, Luiz Gabriel Xavier Botelho, Ronan Christian Machado dos Santos, Isadora Ramos-Andrade, Luiz Guilherme Kraemer-Aguiar, Christina Barja-Fidalgo, João Alfredo Moraes, Mariana Renovato-Martins

**Affiliations:** ^1^ Laboratory of Inflammation and Metabolism, Department of Cellular and Molecular Biology, Universidade Federal Fluminense, Rio de Janeiro, Brazil; ^2^ Redox Biology Laboratory, Programa de Farmacologia e Inflamação, Universidade Federal do Rio de Janeiro, Rio de Janeiro, Brazil; ^3^ Institute of Biophysics, Universidade Federal do Rio de Janeiro, Rio de Janeiro, Brazil; ^4^ Department of Radiation Oncology, University of Miami Miller School of Medicine, Miami, FL, United States; ^5^ Obesity Unit, Multiuser Clinical Research Center (CePEM), Hospital Universitário Pedro Ernesto, Universidade do Estado do Rio de Janeiro, Rio de Janeiro, Brazil; ^6^ Laboratory of Cellular and Molecular Pharmacology, Institute of Biology Roberto Alcantara Gomes (IBRAG), Universidade do Estado do Rio de Janeiro, Rio de Janeiro, Brazil

**Keywords:** leptin, obesity, adipose tissue, extracellular vesicles, breast cancer

## Abstract

**Introduction:**

Leptin, a key adipokine secreted by adipose tissue (AT), has emerged as a critical mediator linking obesity and breast cancer, both of which are major global health concerns. Elevated leptin levels are detected in the circulation and in extracellular vesicles (EVs) released by adipose tissue, particularly in cases of obesity. These leptin-enriched EVs have been implicated in various stages of tumor progression. In this study, we investigated the effects of leptin within extracellular vesicles (EVs) secreted by obese adipose tissue on the functional properties and metabolism of MDA-MB-231 breast cancer cells, a model for triple-negative breast cancer (TNBC).

**Method:**

MDA-MB-231 cells were treated with EVs derived from the subcutaneous adipose tissue of eutrophic (EUT EVs) and obese (OB EVs) individuals.

**Results:**

Our findings revealed that OB EVs induced significant phosphorylation of STAT3, a key signaling molecule in cancer progression, and promoted increased cell migration, dependent on fatty acid oxidation (FAO). This effect was reversed in the presence of a leptin receptor antagonist, highlighting leptin’s pivotal role in these processes. Additionally, OB EVs caused metabolic changes, including reduced lactate levels and decreased pyruvate kinase (PK) activity, while increasing glucose-6-phosphate dehydrogenase (G6PDH) activity, suggesting metabolic reprogramming that supports tumor cell survival and proliferation. In addition to metabolic alterations, OB EVs also impacted mitochondrial dynamics. We observed an upregulation of fusion and fission markers and a redistribution of mitochondria toward the cell periphery, which supports migration. Moreover, OB EVs increased the invasive capacity of MDA-MB-231 cells, an effect mediated by matrix metalloproteinase-9 (MMP-9).

**Discussion:**

Overall, our results highlight how obese adipose tissue modulates breast cancer cell behavior, with leptin-enriched EVs playing a central role in driving migration, metabolic reprogramming, and invasiveness, thereby promoting tumor malignancy. This study underscores the importance of EVs in the obesity-cancer link and offers new insights for therapeutic strategies targeting leptin signaling and EV-mediated communication in breast cancer.

## 1 Introduction

Breast cancer, the second most prevalent malignant neoplasia globally, is a significant health concern, mainly among women, accounting for 11.6% of all cancer cases (1). Obesity, a widespread concern affecting nearly 1 billion people globally (2), has emerged as a significant risk factor for breast cancer, with powerful associations (3) and an increased risk for triple-negative breast cancer (TNBC) in postmenopausal women (4). It is known mainly that obesity, defined by a body mass index (BMI) ≥ 30 kg/m², is linked to an elevated incidence of breast cancer (3) and worsened clinical outcomes, including the development of higher-grade tumors (5), increased metastasis (6), and resistance to chemotherapy (7).

The connection between obesity and breast cancer development is primarily attributed to the biological changes in adipose tissue (AT) accompanying its expansion. As adipose tissue grows, it undergoes complex changes, including chronic low-grade inflammation characterized by immune cell infiltration (8) and the secretion of pro-inflammatory adipokines (9). Among these adipokines, leptin stands out as a central player in promoting breast cancer progression, contributing to key tumorigenic processes, including cell growth, survival (10), migration, and invasion (10). Furthermore, leptin has been shown to impact cancer cell metabolism (11–13) significantly and mitochondrial dynamics (11). Notably, elevated levels of leptin are found in the serum (12), AT secretome (13), and extracellular vesicles (EVs) derived from AT of obese individuals (14).

EVs have garnered increasing attention as essential mediators of intercellular communication within the tumor microenvironment. These vesicles facilitate bidirectional signaling between tumor cells and surrounding tissues, including distant sites, thereby contributing to the overall progression and metastasis of cancer (15). Studies have demonstrated that AT-derived EVs play a pivotal role in promoting various cancer hallmarks (16), including enhanced migratory capacity (17), through the transfer of fatty acid oxidation (FAO) enzymes and substrates, as well as the modulation of mitochondrial dynamics (18). Furthermore, it has been shown that EVs derived from the conditioned media of *in vitro*-differentiated adipocytes can induce phenotypic changes associated with epithelial-to-mesenchymal transition (EMT) (19). Notably, EVs isolated from the breast AT of overweight and obese women have been shown to impact cancer cell malignancy significantly, promoting increased proliferation and mitochondrial respiration (20).

In our previous work, we identified that EVs derived from AT explants of obese individuals enhance the migratory and invasive properties of TNBC cells, with these EVs notably enriched in leptin (14). Based on these findings, the present study investigates the role of leptin within obese AT-derived EVs and its contribution to the malignancy of TNBC, specifically focusing on how metabolic reprogramming underpins aggressive tumor behavior and mitochondrial dynamics. Our results propose a mechanism for leptin delivery via EVs, offering a significant advancement in understanding how EVs released by obese AT act in promoting breast cancer malignancy and providing new insights into the interplay between obesity, leptin signaling, and tumor progression.

## 2 Materials and methods

### 2.1 Participants

This study involved two participant groups: an obese group (OB) and an eutrophic group (EUT). The OB group comprised individuals with obesity who were clinically selected for bariatric surgery, while the EUT group included lean individuals undergoing either mammary plastic surgery or laparoscopic cholecystectomy.

### 2.2 EVs isolation

**Figure 1:**
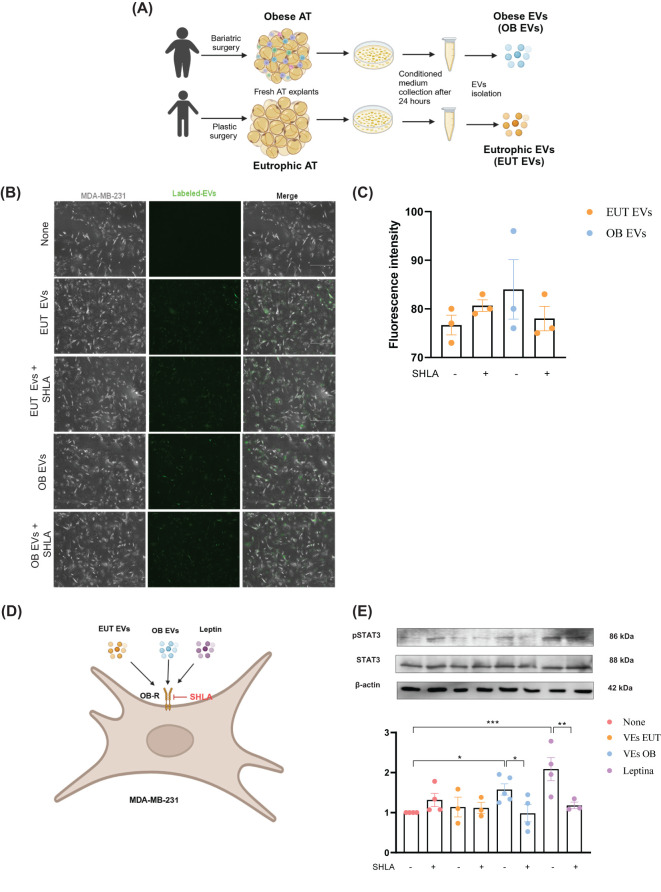
EUT EVs and OB EVs colocalize with MDA-MB-231 cells, and OB EVs activate STAT3 in a leptin-dependent manner. MDA-MB-231 cells were treated with 20% extracellular vesicles derived from eutrophic (EUT EVs) or obese (OB EVs) adipose tissue, in the presence or absence of 250 ng/mL of human super-active leptin antagonist (SHLA). **(A)** Schematic overview of EUT EV and OB EV isolation. Created with BioRender. **(B)** EVs were labeled with the cell tracker green CMFDA dye before cell treatment. Colocalization of EVs with MDA-MB-231 cells was assessed by fluorescence microscopy in the presence or absence of SHLA. **(C)** Quantification of EV fluorescence intensity was performed using ImageJ software. **(D)** Schematic representation of the MDA-MB-231 treatment protocol. **(E)** Total protein extracts from treated cells were subjected to SDS-PAGE and immunoblotting to detect phosphorylated STAT3 (pSTAT3), total STAT3, and actin (loading control). Densitometric analysis was performed using ImageJ to determine the pSTAT3/STAT3 ratio. Results represent 3–4 independent experiments and are expressed as mean ± SEM. Statistical significance was assessed by one-way ANOVA followed by Sidak’s *post hoc* test. *Significance: *p < 0.05, **p < 0.01, ***p < 0.005.

As previously described, the EVs released by the subcutaneous AT explants were obtained (13). Briefly, subcutaneous OB and EUT AT fragments were collected during surgeries, cleaned with PBS, cut into small pieces, and cultivated in M199 (Gibco, 11043023) supplemented with 1% of fetal bovine serum (FBS) (Gibco, 12657-029) in a proportion of 100 mg/mL, for 24 hours (37° C, 5% CO2). Then, conditioned media (CM) were collected, filtered, and centrifuged for 10 min at 350 g/4°C to remove cellular debris. After, it was subjected to two centrifugations: the first at 2,000x g for 10 minutes at 4°C and the second at 20,000x g for 70 minutes at 4°C and the EV-enriched pellet was resuspended in incomplete DMEM F-12 (Gibco, 11330-032) and stored at -4°C for further analysis (**Figure 1A**).

### 2.3 Cell culture

The human breast adenocarcinoma cell line MDA-MB-231 was obtained from the American Type Culture Collection Cell Bank (ATCC). It was maintained in a mixed culture (50% v/v) medium of DMEM F-12 and DMEM high glucose (Gibco, 10569-010) supplemented with 10% FBS, penicillin, and streptomycin (Gibco, 15140122). The cells were cultured at 5% CO2, pH 7.2, and 37°C and were allowed to grow until confluency was reached and detached for use after a brief treatment with trypLE (Gibco, 12605028). After, the cells were centrifuged at 400 x g, at 20°C, for 10 minutes. Cells were used up to the 10th passage at most.

### 2.4 Cell treatment

MDA-MB-231 cells were seeded in plates and treated with 20% (v/v) of EVs derived from EUT (EUT EVs) or OB (OB EVs) AT (13), 100 nM of recombinant human leptin (Leptin) (300-27, Peprotech), and 250 mg/mL of human super leptin antagonist (MyBioSource, MBS400098) (SHLA). Besides that, cells were also treated with 5 µM of etomoxir (Sigma-Aldrich, E1905), 5 Mm of 2-deoxy-D-glucose (2-DG) (Sigma-Aldrich, D3179), 1 mM of palmitate (Supelco, 90930), and LY294002 (Abcam, ab120243). All stimuli were diluted in DMEM F-12, except for the treatment with etomoxir, which was in a mixed culture medium 50% v/v of DMEM F-12 and DMEM high glucose, due to a morphological alteration and reduction in cell viability (**Supplementary Figures 1A, B**). Furthermore, this mixture was also used in the clonogenicity assay due to the long duration of the test.

### 2.5 EVs staining

MDA-MB-231 cells were seeded in glass coverslips and then treated for 1 hour with EUT EVs and OB EVs labeled with 10µM of cell tracker green CMFDA (Invitrogen, C7025) in the presence and absence of SHLA. Cells were pre-warmed with pre-warmed PBS, fixed with 3.7% paraformaldehyde for 15 min at room temperature, and permeabilized with 0.2% Triton X-100 for 5 min. It was imaged at 20x magnification using immunofluorescence microscopy (EVOS™ M7000), and analyses were performed with ImageJ software (NIH).

### 2.6 Immunoblotting

MDA-MB-231 cells were seeded in 12-well plates and treated with stimuli. Then, it was washed with PBS and lysed in RIPA lysis buffer (Sigma-Aldrich, Cat.R0278) with a protease inhibitor cocktail (Roche, 04693116001). BCA (Thermofisher Scientific, Cat.23227) determined protein concentration. Cell extracts were denatured in a buffer (BioRad, Cat.1610737) for 3 minutes at 90°C. 30 µg of protein were loaded onto electrophoresis gels, and the proteins were transferred to nitrocellulose or PVDF membrane. Membranes were blocked with 5% of nonfat dry milk (BioRad, 1706404) for 1 hour at room temperature, incubated with primary antibodies at 4°C overnight, and with peroxidase-conjugated secondary antibody for 1 hour. The following antibodies were used: STAT3 (1:1000; Cell Signaling; 4904S), Phospho-STAT3 Tyr705 (1:1000; Cell Signaling; 9131S), and Actin (1:1000; Abcam; ab119716). For phospho-protein detection, membranes were first incubated with phospho-protein antibody and stripped with Restore PLUS Western blot stripping buffer (Thermo-Fisher Scientific, 46430) for 15 min. Some membranes were cut to allow the visualization of multiple targets with different molecular weights. Bands were visualized using an enhanced chemiluminescent (ECL) (Thermo Scientific, 34095) and quantified by densitometry using ImageJ software (NIH). The results were expressed as arbitrary units.

### 2.7 Cell death analysis

MDA-MB-231 cells were seeded in 12-well plates; after adherence, the cells were “starved” for 12 hours and treated with stimuli for 48 hours. The apoptotic and necrotic cells were measured using Annexin V conjugated with fluorescein isothiocyanate (FITC Annexin V BD, 556419) and Propidium iodide (PI) (BioLegend, 421301) staining (to detect the necrotic cells). The cells were trypsinized, centrifuged at 400 RCF for 8 minutes, washed with cold Annexin V Binding Buffer, and centrifuged again. Next, we resuspended the cells in 50 µL of Annexin V Binding Buffer containing Annexin V [1:200] and PI [1:500] and incubated for 15 min on ice in the dark. Cells were analyzed by flow cytometry using BD FACSCanto™. 10,000 events were acquired per condition. FlowJo v10 software was used for data analysis.

### 2.8 Viability assay

The reduction of thiazolyl blue tetrazolium bromide (MTT) assay analyzed cell viability. MDA-MB-231 cells were seeded in 96-well plates; after adherence, the cells were “starved” for 12 hours. Subsequently, the cells were treated with stimuli for 24 hours, and 5 mg/mL of MTT (Sigma-Aldrich, M5655), diluted in PBS, were added to the cells in the last 4 hours of the stimuli. Formazan crystals formed were dissolved in isopropyl alcohol. Absorbance was detected at 570nm on a DR-200BS-NM-BI plate reader (Kasuaki).

### 2.9 Cell migration assay (wound healing)

Cell migration was assessed using the wound healing method. MDA-MB-231 cells were seeded in 24-well plates until reaching 95% confluence. Cells were washed and treated with mitomycin (5µg/ml) for 2 hours before being washed. Subsequently, a vertical line was made on the plate with a pipette tip to remove the cells. Then, cells were treated with stimuli during the total migration time (24 hours). Such regions were photographed (IV5100 Series Inverted Biological Microscope) at 10x magnification (two photos per field) at 0 h and 24 h. Subsequently, analyses were made using Photoshop software, and cell migration was quantified as a percentage of wound closure compared to the cells at 0 h in each group.

### 2.10 Invasion assay

The invasiveness was analyzed using a transwell chamber containing membranes with µm pores, initially coated with Matrigel-like (Gibco, Cat.A14132-02). MDA-MB-231 cells were seeded and primed for 1 hour. Next, the cells were detached, resuspended, and seeded into the transwell with 1% DMEM. At the lower chamber, 10% DMEM was added as a chemoattractant. After 24 hours of incubation at 37°C and 5% CO_2_, the cells that invaded through the gelatin and remained adhered to the lower part of the membrane of the insert were fixed and stained with a quick panoptic kit (Laborclin). For each experimental group, five random areas, using 20x magnification, were selected, photographed (IV5100 Series Inverted Biological Microscope), and analyzed. Invasive cells were quantified manually using Photoshop software.

### 2.11 Clonogenicity assay

To assess colony formation, MDA-MB-231 cells were seeded in 12-well plates at a density of 250 cells per well, followed by the addition of the stimuli. The medium and the stimuli were replaced every 3 days. After 10 days of treatment, cell colonies were stained with 0.1% crystal violet prepared in 20% methanol. For each experimental group, four random areas were photographed under 10x magnification (IV5100 Series Inverted Biological Microscope) and analyzed. Colonies quantification was performed manually using Photoshop software.

### 2.12 Metalloproteinase-9 activity

Gelatin zymography was performed for EVs derived from MDA-MB-231 cells previously treated with stimuli for 1 hour. After treatment, MDA-MB-231 cells supernatants were collected, and EVs were isolated from them and resuspended in lysis buffer (50 mM HEPES buffer, pH 7.5 containing 250 mM NaCl, 1% Triton X-100, and 10% glycerin), supplemented with protease inhibitors. Then, they were subjected to SDS-PAGE with gels copolymerized with 1 mg/mL gelatin, washed in renaturation buffer (2.5% Triton X-100 in 50 mM Tris–HCl, pH 7.5), and incubated at 37°C for 72 h in substrate buffer (10 mM Tris–HCl buffer, pH 7.5; 5 mM CaCl2; and 1 mM ZnCl2). The gels were stained with Coomassie Brilliant Blue R-250 staining solution (Bio-Rad, Cat.1610435) and destained using Coomassie Brilliant Blue R-250 destaining solution (Bio-Rad, Cat.1610438). Areas of enzymatic activity appeared as clear bands and were measured by densitometry using ImageJ software (NIH). For analysis, the image was digitally inverted.

### 2.13 ATP content assay

MDA-MB-231 cells were seeded in 12-well plates, treated with stimuli for 30 minutes, and lysed. The intracellular ATP content was detected sequentially using a commercial kit (Sigma, MAK190) by colorimetry, according to the manufacturer’s instructions.

### 2.14 Glucose-6-phosphate dehydrogenase activity assay

MDA-MB-231 cells were seeded in 12-well plates and treated with stimuli for 24 hours. Then, cells were lysed, and G6PDH activity was detected using a commercial kit (Sigma, MAK015), using colorimetry, according to the manufacturer’s instructions.

### 2.15 Lactate content assay

MDA-MB-231 cells were seeded in 12-well plates, primed with stimuli for 1 hour, washed, and incubated with 0.25% DMEM for 24 hours. The supernatant was collected, and the cells were lysed. The intracellular and extracellular l-lactate content was detected sequentially using a commercial kit (Sigma, MAK329) by colorimetry, according to the manufacturer’s instructions.

### 2.16 Pyruvate kinase activity assay

MDA-MB-231 cells were seeded in 12-well plates and treated with stimuli for 24 hours. Then, cells were lysed, and PK activity was detected using a commercial kit (Sigma, MAK071) by colorimetry, according to the manufacturer’s instructions.

### 2.17 Mitotracker staining

MDA-MB-231 cells were seeded in glass coverslips and treated with stimuli for 48 hours for mitochondrial analysis. Cells were then incubated with MitoTracker Red CMXRos (Cell Signaling, 9082) (100 nM in PBS, for 45 minutes, at 37°C). Cells were washed three times with pre-warmed PBS, fixed with 3.7% paraformaldehyde for 15 min at room temperature, and permeabilized with 0.2% Triton X-100 for 5 min. After, cells were incubated with ActinGreen 488 (Invitrogen, R37110) for 30 minutes and DAPI (Invitrogen, P36941) for 5 minutes. It was imaged at 40x magnification immediately using confocal microscopy, and analyses were performed using ImageJ software (NIH).

### 2.18 q-RT-PCR

MDA-MB-231 cells were seeded in 12-well plates and treated with stimuli for 24 hours. Then, mRNA was isolated using the RNeasy Plus Kit (Qiagen, 74134) as recommended by the manufacturer. RNA purity and concentration were quantified with an L-quant (Loccus) instrument. cDNA was synthesized with a high-capacity cDNA reverse transcription kit (Applied Biosystems, 4368814) by following the protocol. qPCR was performed with TaqMan™ Fast Advanced Master Mix (Applied Biosystems, 4444557) following manufacturer instructions. The primers used were OPA1- Hs01047013_m1 (Applied Biosystems, 4331182), DNM1L- Hs01552605_m1 (Applied Biosystems, 4331182), and eukaryotic 18S rRNA - Hs99999901_s1 (Applied Biosystems, 4333760T) as a housekeeping gene.

### 2.19 Statistical analysis

Data are expressed as means ± standard error (S.E.). Data normality was checked using the Shapiro-Wilk test. For normal data, the means for quantitative variables between groups were compared using a one-way ANOVA test, followed by the Sidak *post-hoc* test. For non-normal data, the *Kruskal-Wallis* test was performed. For all analyses, a p-value <0.05 was considered statistically significant. Statistical evaluation was performed using GraphPad Prism software version 8.0 (GraphPad Software, La Jolla, CA).

## 3 Results

### 3.1 Extracellular vesicles from obese adipose tissue induce leptin signaling in MDA-MB-231 breast cancer cells

Given the increased leptin content in OB EVs compared to EVs previously published by our group (14) and the evidence that MDA-MB-231 cells abundantly express the leptin receptor (Ob-R) (21), we investigated whether the leptin receptor plays a role in the binding and/or internalization of EVs by MDA-MB-231 cells. Our results showed that both EUT and OB EVs colocalized with MDA-MB-231 cells to the same extent; leptin receptor antagonism did not affect this effect (**Figures 1B, C**). We further examined whether leptin in EUT or OB EVs could activate the leptin signaling pathway in the presence or absence of SHLA, as outlined in the flowchart (**Figure 1D**). Our findings revealed that OB EVs increased STAT3 phosphorylation compared to untreated cells, and this effect was reversed in the presence of SHLA, indicating that leptin in OB EVs drives STAT3 phosphorylation. As expected, a similar increase in phosphorylation was observed in cells treated with leptin (**Figure 1E**).

### 3.2 Leptin-enriched OB-derived extracellular vesicles mitigate early apoptosis in MDA-MB-231 breast cancer cells

**Figure 2:**
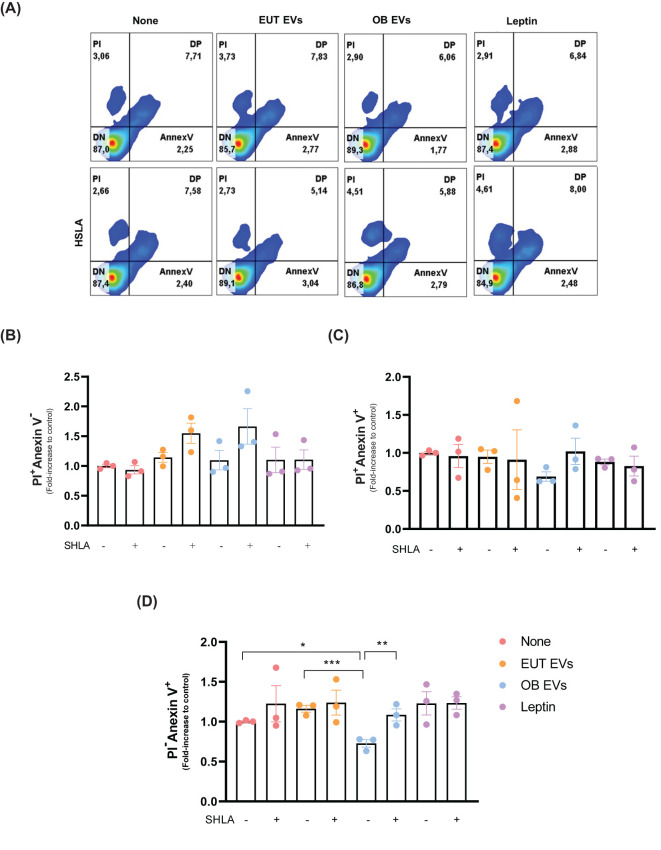
Leptin within OB EVs reduces apoptosis and prevents necrosis in MDA-MB-231 cells. MDA-MB-231 cells were treated with 20% extracellular vesicles derived from eutrophic (EUT EVs) or obese (OB EVs) adipose tissue, 100 nM recombinant leptin, or 250 ng/mL of human super-active leptin antagonist (SHLA). **(A)** Following treatment, flow cytometry was analyzed using Annexin V-FITC and propidium iodide (PI) staining to distinguish between necrotic and apoptotic populations. Quantification of **(B)** necrotic cells (Annexin V^-^/PI^+^), **(C)** late apoptotic cells (Annexin V^+^/PI^+^), and **(D)** early apoptotic cells (Annexin V^+^/PI^-^) was conducted. Results represent three independent experiments and are presented as mean ± SEM. Statistical analysis was performed using one-way ANOVA followed by Sidak’s *post hoc* test. *Significance: *p < 0.05, **p < 0.01, ***p < 0.005.

Given the well-established association between STA3 and tumor progression (22), we investigated the impact of leptin present in OB EVs on key stages of cancer development. To assess this, we first examined the effect of leptin in OB EVs on cell death under serum deprivation conditions. Notably, after 24 hours of serum deprivation, no significant changes in cell viability were observed across the treatment groups (**Supplementary Figure 2**). At 48 hours, 80% of the cells remained viable, regardless of treatment (**Figure 2A**). Among the cells that underwent cell death, the treatments did not alter the proportion of cells undergoing necrosis (**Figure 2B**) or late apoptosis (**Figure 2C**). However, treatment with OB EVs reduced the percentage of early apoptotic cells by 20% and 40% compared to untreated and EUT EVs, respectively. This effect was reversed in the presence of SHLA, highlighting the crucial role of leptin. Interestingly, leptin alone did not replicate this effect (**Figure 2D**), suggesting that additional components in OB EVs are necessary for leptin to exert its full impact.

### 3.3 Leptin-enriched extracellular vesicles from obese cells enhance metastatic properties in MDA-MB-231 breast cancer cells

**Figure 3:**
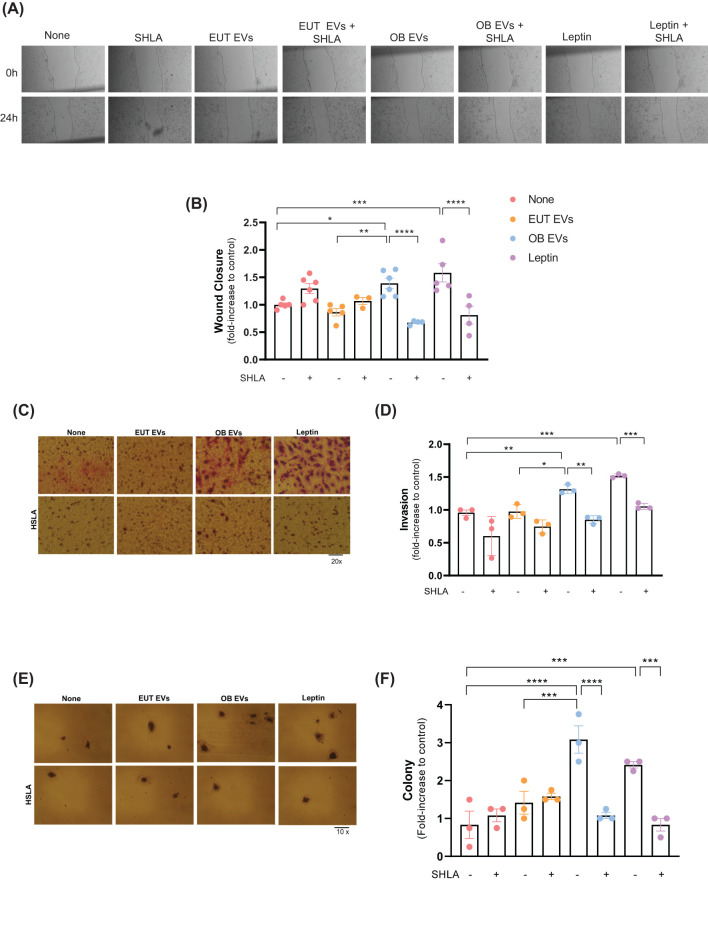
Leptin in OB EVs enhances migration, invasion, and colony formation in MDA-MB-231 cells. MDA-MB-231 cells were treated with 20% extracellular vesicles derived from eutrophic (EUT EVs) or obese (OB EVs) adipose tissue, 100 nM recombinant leptin, and/or 250 ng/mL of the human super-active leptin antagonist (SHLA). **(A)** Cell migration was assessed using the Wound Healing assay 24 hours post-treatment (compared to 0 h). Images were captured using an inverted microscope at 10× magnification (two images per field). **(B)** Quantification of wound closure was performed using Adobe Photoshop. **(C)** Cell invasion was evaluated using a Matrigel-coated Transwell assay. Cells that migrated through the 8 µm-pore membrane were fixed and stained using the panoptic staining kit. **(D)** Migrated cells were counted across five random fields per insert under a 20× magnification using an inverted microscope. Quantification was done using Photoshop CS5. **(E)** Clonogenic potential was assessed via colony formation assays, and **(F)** colony counts were obtained from four random areas per condition. Data represent 3 to 6 independent experiments and are normalized to untreated controls. Statistical analysis was performed using one-way ANOVA with Sidak’s *post hoc* test. Bar graphs represent mean ± SEM. *Significance: *p < 0.05, **p < 0.01, ***p < 0.005, ****p < 0.001.

Building upon the understanding that leptin facilitates the migration and invasion of cancer cells through the JAK2/STAT3 signaling pathway (23), we investigated the influence of leptin within OB EVs on these critical stages of tumor metastasis. Our results revealed that OB EVs significantly enhanced the migration of MDA-MB-231 cells by 40% and 50% compared to untreated cells or cells treated with EUT EVs, respectively. Importantly, this effect was abolished entirely in the presence of SHLA, confirming its role in mediating these responses. In addition, leptin treatment similarly enhanced cell migration, with this increase being reversed upon SHLA treatment, as expected (**Figures 3A, B**). Similarly, both OB EVs and leptin substantially enhanced the invasiveness of MDA-MB-231 cells, increasing it by 30% and 50%, respectively. These effects were again negated by SHLA treatment (**Figures 3C, D**). Furthermore, OB EVs and leptin treatment nearly tripled the clonogenic potential of MDA-MB-231 cells, an effect that was reversed with SHLA co-treatment (**Figures 3E, F**). These findings underscore the critical role of leptin contained within OB EVs in promoting key metastasis processes, including cell migration, invasion, and colony formation.

### 3.4 FAO and AKT signaling are essential for leptin-induced migration enhancement

**Figure 4:**
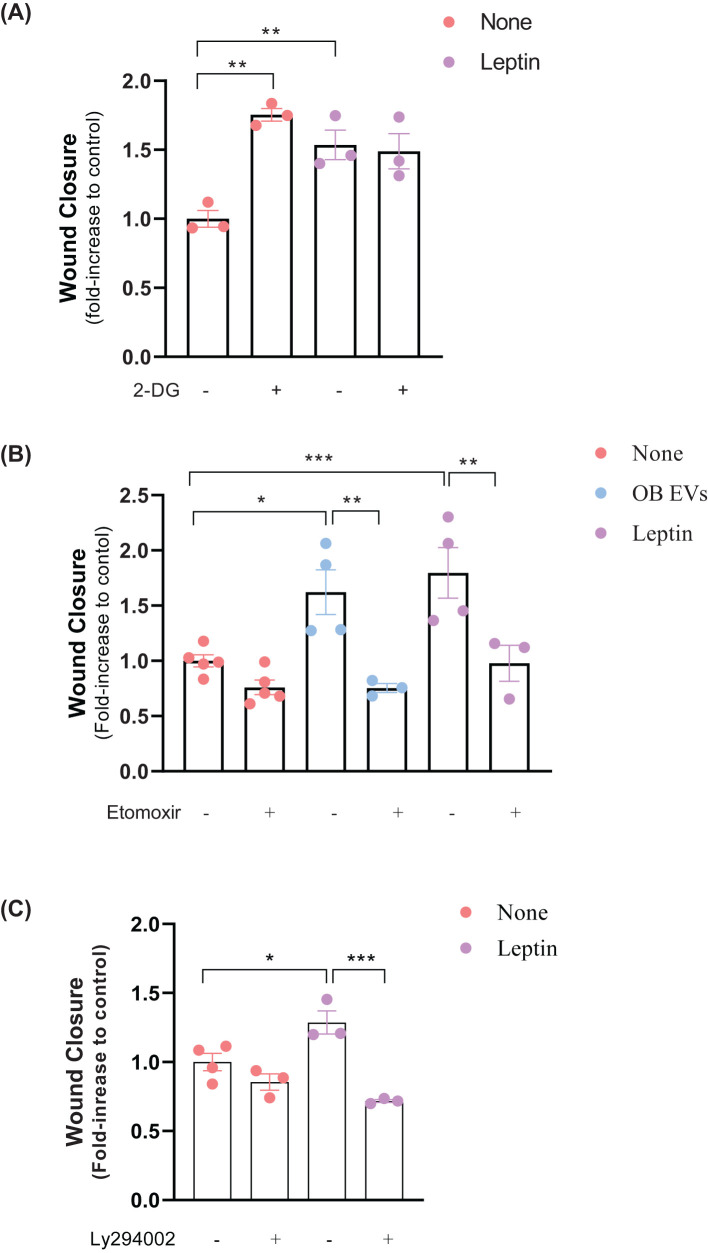
Leptin in OB EVs promotes MDA-MB-231 cell migration via FAO and likely through the AKT signaling pathway. MDA-MB-231 cells were treated with 20% extracellular vesicles from obese adipose tissue (OB EVs) or 100 nM recombinant leptin for 24h. To explore the metabolic and signaling pathways involved, cells were co-treated with 5 mM 2-Deoxy-D-glucose (2-DG) to inhibit glycolysis **(A)**, 50 µM Etomoxir to block fatty acid oxidation **(B)**, or 20 µM LY294002 to inhibit PI3K/AKT signaling **(C)**. Cell migration was assessed by the Wound Healing assay, with images captured at 0 h and 24 h using an inverted microscope at 10× magnification (two images per field). Data represent 3–4 independent experiments and are normalized to unstimulated control cells. Statistical analysis was performed using one-way ANOVA with Sidak’s *post hoc* test. Bar graphs show mean ± SEM. *Significance: *p < 0.05, **p < 0.01, **p < 0.005. the groups. The bar graphs represent means ± standard error. *p<0.05, ***p<0.005.

We examined the role of metabolic alterations to elucidate the mechanisms underlying leptin and OB EVs’ promotion of MDA-MB-231 cell migration. In addition to STAT3 signaling, which is known to contribute to cellular migration, other metabolic processes are essential for this phenomenon. Specifically, we assessed the impact of inhibiting glycolysis and fatty acid oxidation (FAO) on migration. Interestingly, glycolysis (2-DG) inhibition increased migration in untreated cells. However, this effect was not observed in leptin-treated cells, where glycolysis inhibition did not significantly change migratory behavior (**Figure 4A**).

In contrast, FAO inhibition by Etomoxir significantly reversed the increase in migration induced by OB EVs and leptin treatment, underscoring its importance in regulating cell motility (**Figure 4B**). Given the pivotal role of the AKT signaling pathway in reprogramming cancer cell metabolism (27) and its involvement in promoting migration by OB EVs (18), we investigated the effects of inhibiting the AKT pathway on MDA-MB-231 cell migration. The inhibition of AKT by LY294002 signaling did not affect migration in untreated cells. However, it effectively reversed the leptin-induced increase in migration (**Figure 4C**). These results suggest that leptin in OB EVs enhances the migratory capacity of MDA-MB-231 cells through the activation of FAO, likely mediated by the AKT pathway.

Together, these findings highlight the intricate interplay between metabolic reprogramming and signaling pathways in promoting the migratory phenotype of breast cancer cells.

### 3.5 Leptin in OB-derived extracellular vesicles and its potential impact on MMP-9 activity

**Figure 5:**
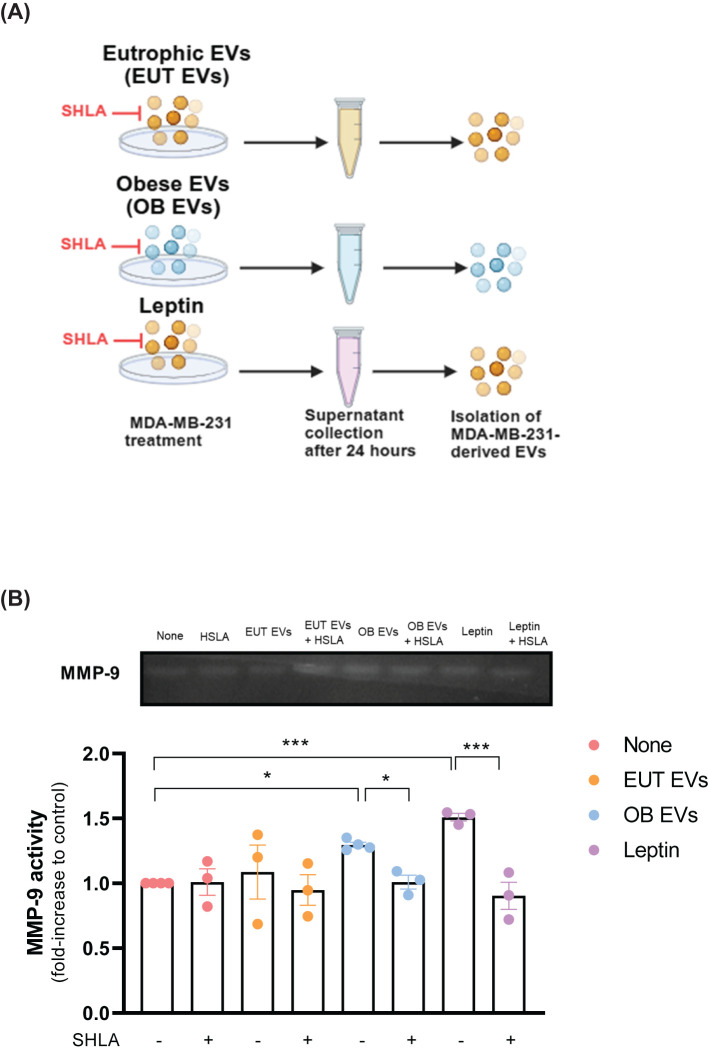
Leptin in OB EVs increases MMP-9 activity in MDA-MB-231 cells. **(A)** MDA-MB-231 cells were primed for 1 hour with 20% extracellular vesicles from eutrophic (EUT EVs) or obese (OB EVs) adipose tissue, 100 nM recombinant leptin, and/or 250 ng/mL human super-active leptin antagonist (SHLA). Following stimulation, cells were maintained in DMEM containing 1% FBS for 24 hours. Supernatants were then collected, and cells were lysed. **(B)** Supernatants were centrifuged at 20,000 × g for 70 minutes at 4°C to isolate EVs. The EV pellets were resuspended in lysis buffer with a protease inhibitor cocktail. Samples were subjected to SDS-PAGE using a gelatin-containing polyacrylamide gel to assess MMP-9 enzymatic activity. Gels were incubated in developing buffer for 72 hours at 37°C, stained with 0.2% Coomassie Brilliant Blue, and destained until clear digestion bands appeared. Band intensity was quantified using ImageJ software. Results are representative of three independent experiments. Statistical analysis was performed using one-way ANOVA with Sidak’s *post hoc* test. Data are presented as mean ± SEM. * Significance: * p < 0.05, *** p < 0.005.

Matrix metalloproteinase 9 (MMP-9) plays a critical role in the proteolytic degradation of extracellular matrix components and can be released within extracellular vesicles by different cancer cell types (24, 25). In this study, we examined MMP-9 activity in EVs isolated from MDA-MB-231 cells treated with EUT EVs, OB EVs, or leptin, with and without SHLA (**Figure 5A**). Our results suggest that EVs from MDA-MB-231 cells treated with OB EVs or leptin contained higher levels of active MMP-9 than untreated cells. This increase in MMP-9 activity was reduced in the presence of SHLA (**Figure 5B**). These findings suggest that leptin, carried by OB EVs, drives this process.

### 3.6 Leptin in OB-derived EVs modulates the metabolic profile of MDA-MB-231 cells

**Figure 6:**
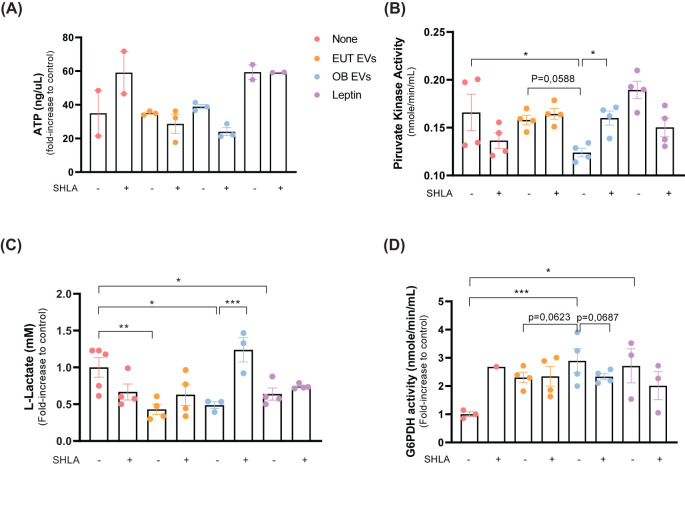
Leptin in OB EVs reduces glycolytic metabolism and enhances pentose phosphate pathway activity in MDA-MB-231 cells. MDA-MB-231 cells were treated with 20% extracellular vesicles derived from eutrophic (EUT EVs) or obese (OB EVs) adipose tissue, 100 nM recombinant leptin, and/or 250 ng/mL of the human super-active leptin antagonist (SHLA). **(A)** After 30 minutes of treatment, intracellular ATP levels were measured using the MAK190 colorimetric assay kit. **(B)** Pyruvate kinase (PK) activity was assessed after 24 hours of treatment using the MAK071 colorimetric kit. **(C)** Cells were primed for 1 hour and cultured for an additional 24 hours before lysis. Lactate production was quantified using the MAK329 colorimetric assay. **(D)** Glucose-6-phosphate dehydrogenase (G6PDH) activity was evaluated in cell lysates after 24 hours of treatment using the MAK014 colorimetric kit. Data represent 3–4 independent experiments and are expressed as mean ± SEM. Statistical significance was determined using one-way ANOVA followed by Sidak’s *post hoc* test. *Significance: *p < 0.05, **p < 0.01, ***p < 0.005.

Cellular metabolism dysregulation is a defining feature of cancer (26), with leptin playing a pivotal role in mediating this alteration (27). Considering this, we explored the impact of leptin within extracellular vesicles on the metabolic processes of MDA-MB-231 cells. Our findings revealed no significant difference in ATP levels among MDA-MB-231 cells treated with EUT EVs, OB EVs, or leptin alone. However, the presence of SHLA led to a noticeable trend towards decreased ATP levels, specifically in cells treated with OB EVs (**Figure 6A**). Although OB EVs did not significantly affect ATP levels, they were found to reduce the enzymatic activity of the glycolytic enzyme pyruvate kinase. This inhibitory effect was reversed in the presence of SHLA in cells treated with OB EVs. Notably, leptin alone failed to replicate this outcome, suggesting that the effect observed with OB EVs may depend on additional substrates or specific components present within the OB EVs (**Figure 6B**).

Further analysis revealed that treatment with EUT EVs, OB EVs, and leptin reduced intracellular levels of lactate, a byproduct of glycolysis. However, SHLA only reversed this effect in MDA-MB-231 cells treated with OB EVs, highlighting a leptin-dependent mechanism specific to OB EVs (**Figure 6C**). These findings suggest that OB EVs suppress glycolytic metabolism through a leptin-dependent pathway. Consistent with this, we observed that OB EVs significantly enhanced the activity of glucose-6-phosphate dehydrogenase (G6PDH), a key enzyme in the pentose phosphate pathway (PPP), in MDA-MB-231 cells. The increase in G6PDH activity was reversed in the presence of SHLA (**Figure 6D**), further supporting the notion that leptin within OB EVs can modulate metabolic flux.

### 3.7 Leptin in OB-derived extracellular vesicles modulates mitochondrial dynamics in MDA-MB-231 cells

**Figure 7:**
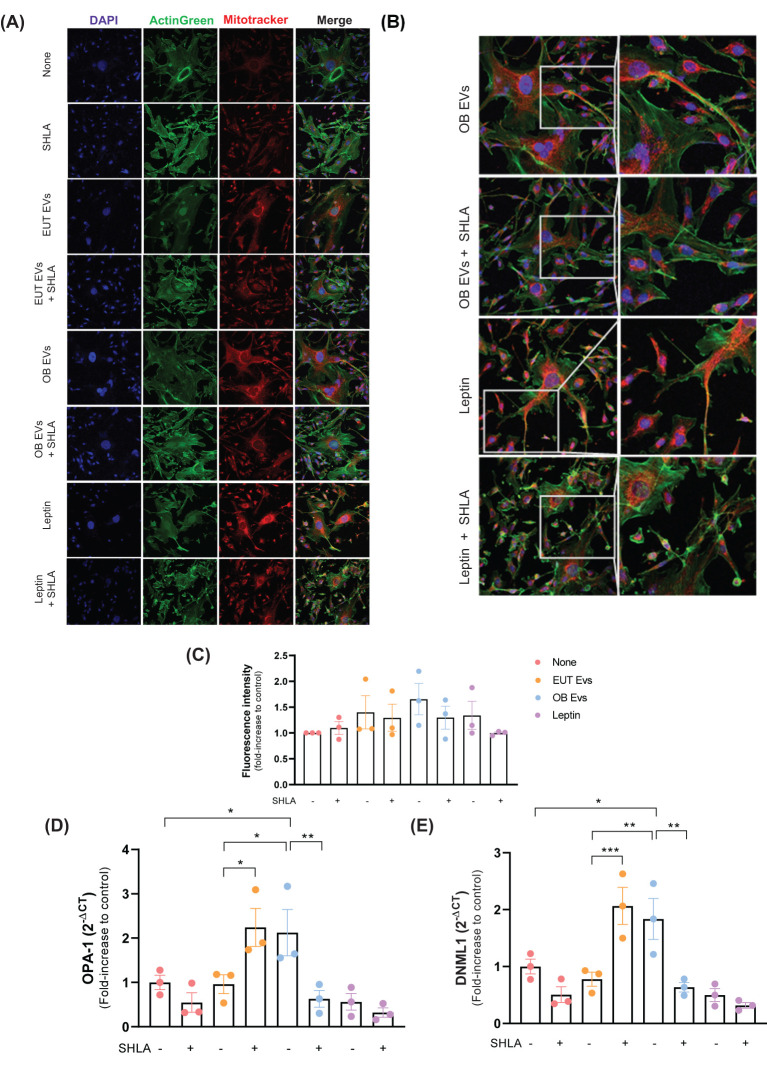
Leptin in OB EVs remodels the mitochondrial network in MDA-MB-231 cells. MDA-MB-231 cells were treated with 20% extracellular vesicles derived from eutrophic (EUT EVs) or obese (OB EVs) adipose tissue, 100 nM recombinant leptin, and/or 250 ng/mL of the human super-active leptin antagonist (SHLA). **(A)** Following treatment, cells were stained with MitoTracker Red CMXRos (mitochondria, red), ActinGreen 488 (actin cytoskeleton, green), and DAPI (nuclei, blue), and imaged using confocal microscopy. **(B)** Mitochondrial fluorescence intensity was quantified using ImageJ. **(C)** Mitochondrial distribution patterns were manually evaluated. **(D, E)** Total RNA was extracted, and quantitative PCR (qPCR) was performed to assess the expression of mitochondrial dynamics-related genes *DNM1L* **(D)** and *OPA1* **(E)**. Data represent three independent experiments and are shown as mean ± SEM. Statistical analysis was performed using one-way ANOVA followed by Sidak’s *post hoc* test. *Significance: *p < 0.05, **p < 0.01, ***p < 0.005.

The dynamic regulation of mitochondrial activity has become increasingly recognized as a crucial driver of tumorigenesis and cancer progression (28). To investigate whether leptin within OB EVs influences mitochondrial function, MDA-MB-231 cells were treated with EUT EVs, OB EVs, or leptin, both in the presence and absence of SHLA. After treatments, cells were labeled with Mitotracker Green to assess mitochondrial morphology, distribution, and activity in response to those treatments. Both groups of cells treated with OB EVs and leptin (**Figures 7A, B**) showed increased mitochondrial fluorescence intensity, suggesting enhanced mitochondrial content. Notably, we observed that OB EVs appeared to induce a redistribution of mitochondria, which were colocalized in membrane protrusions; this effect was reversed upon the addition of SHLA. A similar redistribution was also observed in cells treated with leptin alone (**Figure 7C**), further highlighting the role of leptin in modulating mitochondrial dynamics.

Furthermore, OB EVs led to a significant increase in the expression of two key regulators of mitochondrial fission and fusion, dynamin 1-like (DNML-1), the gene encoding dynamin-related protein 1 (Drp1), and optic atrophy 1 (OPA-1) (**Figures 7D, E**). These findings suggest that OB EVs may modulate mitochondrial fission and fusion processes. The upregulation of these markers was reversed in the presence of SHLA, highlighting the critical role of leptin in regulating mitochondrial dynamics within OB EVs. Interestingly, leptin alone did not replicate this effect, suggesting that leptin acts synergistically with other components present in OB EVs to sustain this dynamic modulation. In contrast, treatment with EUT EVs had no significant impact on DNML-1 or OPA-1 expression. However, blocking leptin action with SHLA led to an unexpected increase in both markers of mitochondrial dynamics, pointing to a complex interplay between SHLA and mitochondrial regulation (**Figures 7D, E**).

## 4 Discussion

EVs have emerged as essential mediators of intercellular communication, playing a pivotal role in cancer progression (15, 29). They facilitate bidirectional communication between different cell types within the tumor microenvironment (TME), influencing cancer cell behavior, metastasis, and tumor growth (15). Notably, AT-derived EVs have garnered significant attention for their substantial contribution to cancer progression. These EVs promote several key hallmarks of cancer, such as enhanced cell proliferation, migration, and invasion (16), and are also elevated in circulation during obesity (13, 30). Our previous work demonstrated that TA-derived EVs enhanced the migration and invasiveness of MDA-MB-231 cells. Furthermore, we found that the AT-derived EVs contained a higher leptin content than those from lean AT (14).

Leptin, which is present at elevated levels in the circulation of individuals with obesity (34), plays a key role in modulating critical cellular processes in tumor cells, including proliferation, metastasis, apoptosis inhibition, angiogenesis, chemoresistance (31), and metabolic reprogramming (27). Upon binding to its receptor, Ob-R, leptin activates a cascade of intracellular signaling pathways, such as JAK2/STAT3, MAPK, and PI3K/AKT (32), which regulate these processes (33). Ob-R is overexpressed in breast cancer cells (34), including the MDA-MB-231 cell line (35). While leptin is widely recognized as a molecular link between obesity and breast cancer, the specific role of leptin within EVs remains underexplored. To address this gap, we investigated how leptin within AT-derived EVs influences the tumorigenic potential of MDA-MB-231 cells. This study proposes a novel mechanism linking obesity, AT-derived EVs, and leptin to breast cancer progression.

A key mechanism in EVs mediating intercellular communication is targeting and anchoring to the plasma membrane of the recipient cell (36). This process can involve activating cell surface receptors, internalizing EVs via endocytosis, or the direct fusion with the cell membrane (37). Alternatively, EVs may undergo lysis in the extracellular space, releasing their contents to stimulate receptors and trigger intracellular signaling (38). Recent studies have highlighted that AT-derived EVs are a significant component of the white adipose tissue secretome (39), with at least 897 exoadipokines identified via proteomics (40). It is well-established that some proteins are carried within the interior of EVs, while others are present on their surface. Regarding leptin localization, there is a lack of direct evidence from the literature. However, it is plausible that most leptin is located within the interior of AT-derived EVs, as our previous study detected leptin in EVs after probe sonication (14). Consistent with this, it was already demonstrated that, although leptin in adipocytes is localized near the plasma membrane, it is packaged inside vesicular structures, as trypsin treatment did not affect its detection, unlike transmembrane proteins such as GLUT4 (41). In contrast, Blandin et al. recently reported that adiponectin is found on the outer membrane of EVs from visceral AT, likely due to nonspecific adsorption of soluble adiponectin (42). Therefore, while it remains speculative, the presence of small amounts of leptin on the EV membrane cannot be completely ruled out.

Our results demonstrated that both EUT and OB EVs interacted similarly with MDA-MB-231 cells, independently of direct OB-R interactions. However, distinct biological effects were observed afterward, such as increased STAT3 phosphorylation induced by OB EVs or leptin. This effect was dependent on OB-R, as it was abolished in the presence of SHLA. These findings suggest that the leptin carried by OB EVs activates OB-R, triggering a cascade of oncogenic processes, including enhanced resistance to apoptosis (52).

Tumor cell expansion depends on increased proliferation and reduced cell death, with resistance to apoptosis playing a pivotal role in sustaining growth (42). Leptin is known to prevent apoptosis through multiple signaling pathways, including PI3K/AKT (43), ERK1/2 (44), and mTOR (45). These pathways inactivate downstream apoptotic factors such as Bad, GSK3, FOXO, and caspase 9, providing pro-survival signals that protect cells from apoptosis (46–48).

To investigate the potential influence of leptin within OB EVs on death, MDA-MB-231 cells were cultured in a serum deprivation medium, a common apoptotic stressor. Under these conditions, OB EVs reduced the percentage of early apoptotic cells compared to untreated or treated with EUT EVs, which was fully reversed in the presence of SHLA. Interestingly, leptin alone did not replicate the anti-apoptotic effect of OB EVs, suggesting that additional molecular components act synergically for leptin to exert its full protective impact.

Leptin’s contribution to tumor cell migration and invasiveness has been documented in various types of cancer, including hepatocellular carcinoma (49), prostate cancer (50), and breast cancer (51). Among the mechanisms, one may highlight the JAK2/STAT3 signaling pathway (23), the activation of the extracellular-signal-regulated kinase (ERK) pathway (52), the upregulation of acetyl-CoA acetyltransferase 2 (ACAT2) (53), and the induction of IL-8 expression in tumor-associated macrophages (TAMs) (54).

The role of EVs in the interactions between adipose tissue and TME, triggering cell migration, is critical (55, 56). Lazar et al. observed that adipocyte-derived exosomes promoted melanoma cell migration (17), and Lin et al. demonstrated that ADMSC-derived exosomes increased the migration of breast cancer cells (57). In the context of obesity, Ramos-Andrade et al. reported a significant role of OB EVs in increasing the migratory capacity of MDA-MB-231 cells (14). Our findings align with and further validate these observations, demonstrating that OB EVs substantially boost the migration of MDA-MB-231 cells, an effect abolished in the presence of SHLA, proposing an essential role of leptin in this process. EVs derived from adipocytes have already been reported as modulators of increased cell migration dependent on FAO. This mechanism occurs due to the delivery of key FAO-related enzymes (17). Our data reveal that FAO inhibition reverses the enhanced migration induced by OB EVs and leptin, emphasizing the critical role of FAO in regulating MDA-MB231 cell migration. In contrast, glycolysis does not seem to be essential in this specific context, as its inhibition had no significant effect.

Further supporting this, the inhibition of AKT signaling in MDA-MB-231 cells effectively reversed the pro-migratory effect of leptin. This is consistent with the study of Kato et al., who identified AKT signaling as essential for leptin-stimulated migration (58) and our previous findings showing that OB EV–driven migration depends on AKT activation. Given that AKT is a central regulator of cancer cell metabolism (59), these results suggest it is a key molecular link connecting leptin signaling, FAO, and enhanced tumor cell migration.

In addition to migration, OB EVs increased the invasiveness of MDA-MB-231 cells in a leptin-dependent manner. These findings align with established literature demonstrating leptin’s ability to stimulate cancer cell invasiveness (60, 61) and the contribution of the AT microenvironment in facilitating these processes (56, 62).

Crucial in cell invasion, one may highlight proteolytic enzymes (63), such as the MMPs. We propose that MMP-9 plays a role in this effect, as its activity was partially reduced by inhibiting the leptin receptor. These findings align with previous studies showing that leptin signaling can upregulate MMPs, including MMP-2, MMP-9 (64), and MMP-13 (61), mainly through STAT3 activation (22). For instance, Lee et al. demonstrated that enhanced invasion is closely associated with the upregulation of MMP-9 (62). Similarly, it has been shown that exosomes derived from 3T3-L1 differentiated adipocytes increased the invasive capacity of lung tumor cells by activating MMP-9, a process driven by the transfer of MMP-3 (65), and that OB EVs exhibit elevated levels of MMP-9, which were transferred to MDA-MB-231 cells (14).

Finally, leptin within OB EVs also promoted increased clonogenicity of MDA-MB-231 cells, supporting the findings of Knight et al., who demonstrated leptin’s role in promoting clonogenicity in cancer cells (66). Collectively, these results highlight the critical role of the leptin derived from the obese AT within EVs in driving the increased malignancy of breast cancer cells.

Metabolic reprogramming has long been recognized as a hallmark of cancer (26), as tumor cells exhibit a remarkable degree of metabolic plasticity (67), allowing them to adapt their energy pathways in response to environmental changes (68). This adaptability is critical for tumor progression, as cancer cells must maintain a high-energy state and produce biosynthetic precursors to sustain their elevated proliferation rates (69). Among the various metabolic pathways, oxidative metabolism has emerged as a key contributor to the aggressive behavior of tumor cells (27, 70, 71), challenging Otto Warburg’s classic view that tumor cells predominantly rely on glycolysis (72).

Consistently, our findings indicate a shift away from the Warburg effect, as OB EVs reduce glycolytic activity and redirect intermediates toward the PPP in MDA-MB-231 cells via a leptin-dependent mechanism - evidenced by decreased PK activity and lactate levels, alongside increased G6PDH activity. Notably, leptin alone did not reproduce this effect, suggesting that other bioactive components in OB EVs contribute to the metabolic reprogramming. Unpublished data from our group show that OB EVs are enriched in palmitic acid and fatty acid binding protein 4 (FABP4), a molecule known to promote breast cancer progression through the IL-6/STAT3/ALDH1 pathway (73). FABP4 has also been implicated in metabolic regulation, potentially linking obesity to breast cancer risk (74).

Previous studies have highlighted the role of adipocyte-derived EVs in promoting FAO and mitochondrial respiration, thereby enhancing ATP production (17, 18). Leptin has similarly been shown to stimulate FAO and oxidative phosphorylation (OXPHOS) via pathways such as c-Myc/PGC-1 (27) and AMPK (75). Interestingly, although our findings indicate a leptin-dependent suppression of glycolysis by OB EVs, ATP levels in MDA-MB-231 cells remained unchanged 30 minutes after the treatment. It aligns with observations by Pharm et al., who reported that leptin increases ATP levels in a time- and dose-dependent manner in MCF-7 and T47D cells, but not in MDA-MB-231 cells, pointing to a potential role for estrogen receptor (ER) signaling in mediating leptin-driven ATP production (76).

Highlighting the metabolic reprogramming driven by OB EVs in MDA-MB-231 cells, our results revealed increased phosphorylation of ACC, a hallmark of FAO activation, which was reversed by etomoxir treatment (**Supplementary Figure 4**). This finding aligns with previous work by Rios Garcia et al., who reported leptin-induced ACC phosphorylation in breast cancer cells (77). Concurrently, both OB EVs and leptin reduced phosphorylation of ATP citrate lyase (ACLY), an effect also reversed by etomoxir (**Supplementary Figure 3**). Given ACLY’s role in converting citrate to acetyl-CoA, a substrate for ACC in the lipogenic pathway (78), these data further support the idea that OB EVs and leptin suppress glycolysis while promoting FAO.

Mitochondria are key metabolism and cell death regulators, constantly adapting to cellular needs through fusion and fission processes. Fusion is driven by mitofusin-1 (MFN1), mitofusin-2 (MFN2), and OPA1, while fission is controlled by Drp1, mitochondrial fission 1 protein (FIS1), mitochondrial fission factor (MFF), and mitochondrial elongation factor 1 (MIEF1). These proteins also coordinate the redistribution of mitochondria toward areas of high energy demand, optimizing energy production (79, 80). Notably, MFN2, OPA1, DRP1, and FIS1 have been found to be overexpressed in oncocytic tumors, independent of mitochondrial content (81).

Adding to this understanding, Liu et al. showed that AT-derived EVs increase Drp1 and MFF in breast cancer cells, without affecting OPA1 (20).

Our study shows that OB EVs upregulate DNML-1 and OPA-1, highlighting their role in modulating mitochondrial dynamics. Notably, SHLA reversed these changes. However, leptin alone was insufficient to reproduce the effect, pointing to a synergistic action with other OB EV components. Strikingly, we also observed that OB EVs and leptin promote mitochondrial redistribution toward membrane protrusions, an event crucial in facilitating tumor cell migration (82). This change, which SHLA reversed, mirrors findings by Zhao et al., who reported that mitochondria migrate from perinuclear zones to membrane protrusions during lamellipodia formation, a key step in migration (83). Similarly, Clement et al. demonstrated that adipocyte-derived exosomes drive mitochondrial redistribution in melanoma cells, further linking this phenomenon to enhanced cell motility and metastatic potential (18).

## 5 Conclusion

**Figure 8:**
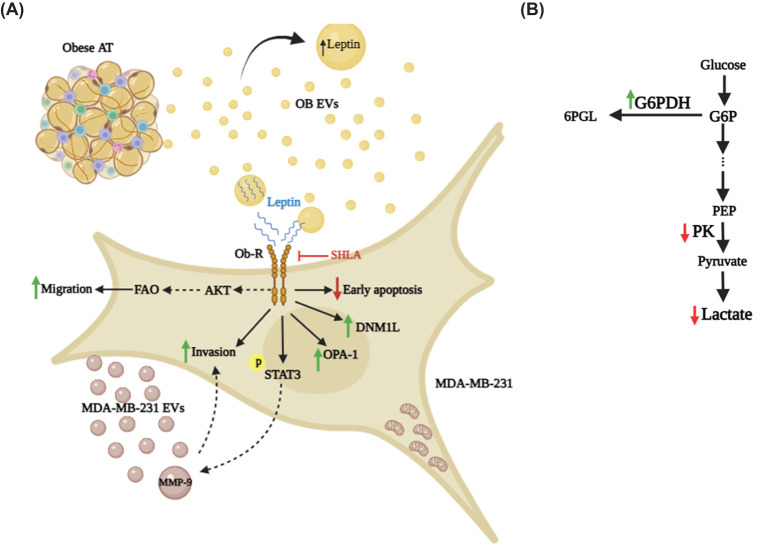
Leptin in OB EVs modulates migration, invasion, clonogenicity, and metabolism of MDA-MB-231 cells. **(A)** Leptin within OB EVs interacts with its receptor (Ob-R) on MDA-MB-231 cells, initiating a cascade of cellular events that significantly enhance cell migration in a fatty acid oxidation (FAO)-dependent manner, likely through activation of the AKT signaling pathway. This interaction also promotes clonogenicity and activates STAT3, a key regulator of oncogenic processes, while increasing the invasive potential of the cells, likely through elevated MMP-9 activity. MMP-9, which is upregulated in EVs released by MDA-MB-231 cells, may be driven by STAT3 activation, further enhancing cancer cell aggressiveness. Additionally, leptin in OB EVs alters mitochondrial dynamics by increasing DNM1-L and OPA-1, redistributing mitochondria toward membrane protrusions, and preventing early apoptosis. **(B)** In addition to its impact on cell motility and survival, leptin in OB EVs induces metabolic shifts in MDA-MB-231 cells. These changes include enhanced G6PDH activity in the pentose phosphate pathway, reduced lactate accumulation, and decreased PK activity. 6PGL, 6-Phosphogluconolactone; AT, adipose tissue; G6P, glucose-6-phosphate; G6PDH, glucose-6-phosphate-dehydrogenase; SHLA, human super leptin antagonist; MDA-MB-231 EVs, extracellular vesicles derived from MDA-MB-231 cells; MMP-9, matrix metalloproteinase 9; OB EVs, extracellular vesicles derived from obese adipose tissue; Ob-R, leptin receptor; PEP, phosphoenolpyruvate; PK, pyruvate kinase.

This study provides a comprehensive and in-depth understanding of the pivotal role of leptin-rich extracellular vesicles derived from obese adipose tissue (OB EVs) in enhancing breast cancer malignancy. By activating leptin receptor (Ob-R)-mediated pathways, these EVs promote tumor progression through increased migration, invasion, and metabolic reprogramming of MDA-MB-231 cells. The findings reveal key mechanisms that support cancer cell survival and adaptability, including FAO- and AKT-dependent migration, STAT3 activation, MMP-9-mediated invasiveness, and mitochondrial remodeling (**Figures 8A, B**).

Crucially, the study positions OB EVs and their leptin content as central players linking obesity to breast cancer progression. This identifies leptin signaling and EV-mediated communication as promising therapeutic targets. Interventions aimed at disrupting leptin-EV pathways or modulating their downstream effects may offer novel strategies for potential therapeutic interventions for mitigating obesity-driven breast cancer aggressiveness.

Yet, the whole picture is still unfolding. Future research is essential to identify other bioactive components within OB EVs that may act synergistically with leptin, and to validate these mechanisms across diverse breast cancer models, including *in vivo* systems. These next steps are critical for translating these insights into impactful, targeted therapies.

## Data Availability

The original contributions presented in the study are included in the article/**Supplementary Material**. Further inquiries can be directed to the corresponding author.
