# Supplementary material for: Targeting the leptin receptor promotes MDA-MB-231 cells’ metabolic reprogramming and malignancy: the role of extracellular vesicles derived from obese adipose tissue
Source: Front Oncol. 2025 May 23;15:1568524. doi: 10.3389/fonc.2025.1568524 (PMC12141216; doi:10.3389/fonc.2025.1568524)
Supplement: Supplementary file 1 [file DataSheet1.docx]

Supplementary Material

#
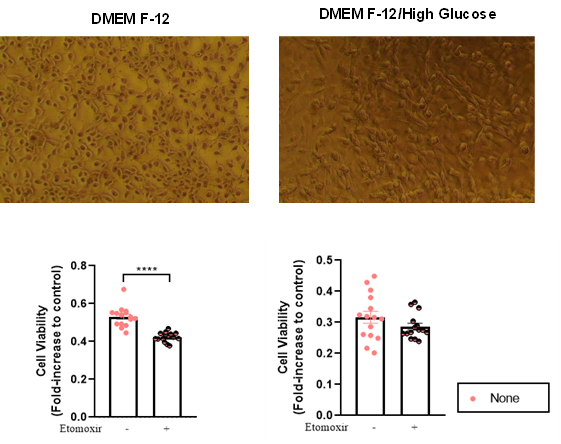
Supplementary Figures


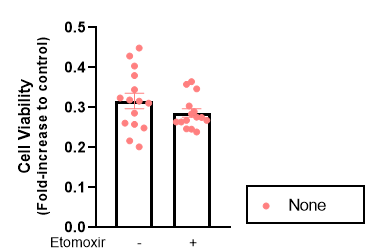

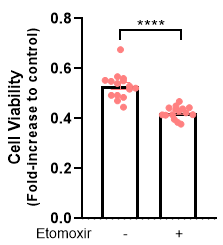


**Supplementary Figure 1.** Etomoxir impacts MDA-MB-231 morphology and viability when treated in DMEM F-12. MDA-MB-231 cells were treated for 24 hours with 50 µM de Etomoxir in incomplete DMEM F-12 and a mixture (50% v/v) of incomplete DMEM-F-12 and DMEM high glucose. (A) Images were obtained with an inverted microscope to an increase of 10x. (B) Cell viability was evaluated using the MTT method. The results are representative of 3 experiments in quintuplicate. A T-test assessed the difference between groups (**** p <0.001). The results were represented as mean ± standard error.

**
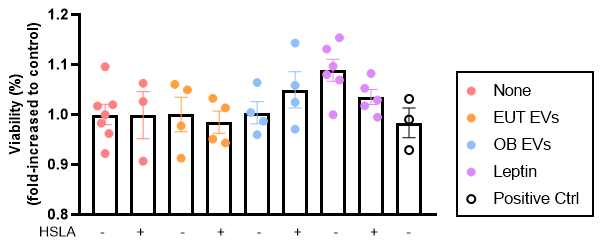
**

**Supplementary Figure 2.** The stimuli do not alter MDA-MB-231 cell viability. MDA-MB-231 cells were treated for 24 hours with 20% EVs from eutrophic (EUT) and obese (OB) AT, 100 nM leptin, and 250 ng/mL of human super-active leptin antagonist (HSLA). DMEM 10% was used as the experiment's positive control (positive CTRL). Subsequently, cell viability was evaluated using the MTT method. The results are representative of 3 to 6 experimental groups. The ANOVA One-Way Post-Test Test was used to assess the difference between the groups, and it was seen that there was no statistically significant difference. The results were represented as an average standard error.


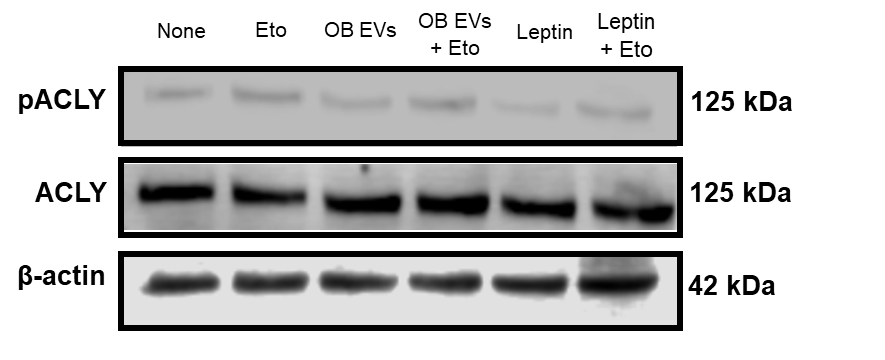


**Supplementary Figure 3.** FAO inhibition with etomoxir (Eto) reduces ATP citrate lyase (ACLY) phosphorylation in MDA-MB-231 cells treated with OB EVs and leptin. MDA-MB-231 was treated with 20% EVs from obese AT (OB EVs), 100 nM leptin, and 5 µM of etomoxir (Eto). The total extract of treated cells was subjected to SDS-PAGE and subsequent immunoblotting to detect Phospho-Acetyl-Coa Carboxylase (pACLY) Ser79 (1:500; Cell signaling, 11818). ATP-Citrate Lyase (ACLY) (1:1000; Cell Signaling, 4332) and Actin (1:1000; Abcam; ab119716) as a loading control. The results are representative of 2 experiments.


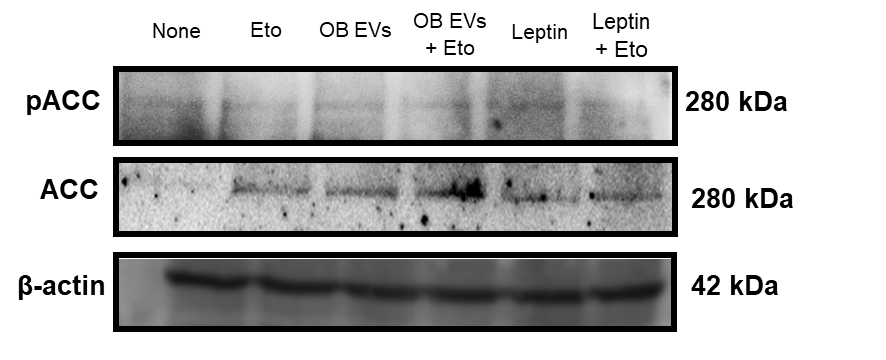


**Supplementary Figure 4.** OB EVs and leptin treatment appear to increase acetyl-CoA-carboxylase (ACC) phosphorylation in MDA-MB-231 cells, and FAO inhibition, with etomoxir (Eto), seems to reduce this phosphorylation. MDA-MB-231 was treated with 20% EVs from obese AT (OB EVs), 100 nM leptin, and 5 µM of etomoxir (Eto). The total extract of treated cells was subjected to SDS-PAGE and subsequent immunoblotting for detection of phospho-acetyl-Coa carboxylase (pACC) Ser79 (1:500; Cell signaling, 11818), acetyl-Coa-carboxylase (ACC) (1:1000; Cell Signaling, 3673), and actin (1:1000; Abcam; ab119716), as a loading control. The result is representative of 1 experiment.
